# *Vital Signs*: Mammography Use and Association with Social Determinants of Health and Health-Related Social Needs Among Women — United States, 2022

**DOI:** 10.15585/mmwr.mm7315e1

**Published:** 2024-04-18

**Authors:** Jacqueline W. Miller, Jessica A. King, Katrina F. Trivers, Machell Town, Susan A. Sabatino, Mary Puckett, Lisa C. Richardson

**Affiliations:** ^1^Division of Cancer Prevention and Control, National Center for Chronic Disease Prevention and Health Promotion, CDC; ^2^Division of Population Health, National Center for Chronic Disease Prevention and Health Promotion, CDC.

## Abstract

**Introduction:**

Approximately 40,000 U.S. women die from breast cancer each year. Mammography is recommended to screen for breast cancer and reduce breast cancer mortality. Adverse social determinants of heath (SDOH) and health-related social needs (HRSNs) (e.g., lack of transportation and social isolation) can be barriers to getting mammograms.

**Methods:**

Data from the 2022 Behavioral Risk Factor Surveillance System were analyzed to estimate the prevalence of mammography use within the previous 2 years among women aged 40–74 years by jurisdiction, age group, and sociodemographic factors. The association between mammography use and measures of SDOH and HRSNs was assessed for jurisdictions that administered the Social Determinants and Health Equity module.

**Results:**

Among women aged 50–74 years, state-level mammography use ranged from 64.0% to 85.5%. Having health insurance and a personal health care provider were associated with having had a mammogram within the previous 2 years. Among women aged 50–74 years, mammography prevalence was 83.2% for those with no adverse SDOH and HRSNs and 65.7% for those with three or more adverse SDOH and HRSNs. Life dissatisfaction, feeling socially isolated, experiencing lost or reduced hours of employment, receiving food stamps, lacking reliable transportation, and reporting cost as a barrier for access to care were all strongly associated with not having had a mammogram within the previous 2 years.

**Conclusions and Implications for Public Health Practice:**

Identifying specific adverse SDOH and HRSNs that women experience and coordinating activities among health care providers, social services, community organizations, and public health programs to provide services that help address these needs might increase mammography use and ultimately decrease breast cancer deaths.

**Summary**

**What is already known about this topic?**

Approximately 40,000 U.S. women die from breast cancer each year. Mammography is recommended to screen for breast cancer and reduce breast cancer mortality. Adverse social determinants of health (SDOH) and health-related social needs (HRSNs) can be barriers to receiving mammograms.

**What is added by this report?**

Mammography use decreased with increasing adverse SDOH and HRSNs experienced. Social isolation, life dissatisfaction, and cost as a barrier to health care access were strongly associated with decreased mammography use.

**What are the implications for public health practice?**

Identifying specific adverse SDOH and HRSNs that women experience, and coordinating activities among health care providers, social services, community organizations, and public health programs to provide relevant services might increase mammography use and ultimately decrease breast cancer deaths.

## Introduction

Each year, breast cancer causes approximately 40,000 deaths among women in the United States (*1*). Although breast cancer death rates (breast cancer deaths per 100,000 women) have been decreasing, this reduction has not been equitable among all populations (*2*). Women who are non-Hispanic Black or African American (Black) and those who have low incomes are more likely to die from breast cancer (*3*,*4*). The U.S. Preventive Services Task Force (USPSTF) currently recommends that women aged 50–74 years have a screening mammogram every 2 years and that women aged 40–49 years should make an informed decision with their health care provider about screening (*5*). These recommendations might change in the near future because the USPSTF recently released a draft recommendation that women aged 40–74 years should have a screening mammogram every 2 years.* Mammograms can detect breast cancers at early stages when they are easier to treat (*6*). During the past decade, several studies have documented that some women were not up to date with receiving a mammogram per recommendations (*7*–*9*).

The U.S. Department of Health and Human Services defines social determinants of health (SDOH) as “conditions where people are born, live, learn, work, play, worship, and age that affect a wide range of health, functioning, and quality-of-life outcomes and risks” (*10*). Health-related social needs (HRSNs) are individual-level, adverse social conditions that can negatively affect a person’s health or health care (*11*). Examples include food insecurity, housing instability, and lack of access to transportation.

Mammography use varies across the United States and is lowest among women without health insurance, those who have low incomes, and those who do not have a usual source of health care (*7*–*9*). These populations typically experience adverse SDOH and HRSNs that serve as barriers to receipt of health care (*12**,**13*). Understanding the impact of certain SDOH and HRSNs on mammography use could help improve cancer control efforts to reduce breast cancer deaths. This study assessed the association between mammography use and a comprehensive list of specific SDOH and HRSNs.

## Methods

### Behavioral Risk Factor Surveillance System

The Behavioral Risk Factor Surveillance System (BRFSS) is an annual, state- and population-based, combined landline and cell phone survey of the civilian, noninstitutionalized adult population aged ≥18 years. BRFSS collects information on health risk behaviors, preventive health practices, health care access, chronic diseases and conditions, and health outcomes across the United States. The median response rate for the 2022 BRFSS across jurisdictions was 45.1% (*14*). SAS-callable SUDAAN (version 9.4; RTI International) was used to analyze all data. This activity was reviewed by CDC, deemed not research, and was conducted consistent with applicable federal law and CDC policy.^†^

### Mammography Use Data

Female BRFSS respondents aged ≥40 years in all states and the District of Columbia (DC) were asked if they had ever had a mammogram, and if so, when they had their last mammogram. Respondents who declined to answer, had a missing answer, or answered, “don't know/not sure” to the mammography questions and those who reported a personal history of breast cancer were excluded from the analysis. Mammography use was analyzed by age group, other demographic characteristics, jurisdiction, and the United States overall. Nonoverlapping 95% CIs were used as a proxy for statistical significance. Data were weighted to the age, sex, and racial and ethnic distribution of each jurisdiction's adult population using intercensal estimates (*15*).

### Social Determinants of Health and Health-Related Social Needs Data

Thirty-nine states^§^ and DC collected data through the new BRFSS Social Determinants and Health Equity module. The adverse SDOH measures were lost or reduced hours of employment (“In the past 12 months have you lost employment or had hours reduced?”), food insecurity (“During the past 12 months how often did the food that you bought not last, and you didn’t have money to get more?”), housing insecurity (“During the last 12 months, was there a time when you were not able to pay your mortgage, rent or utility bills?”), experiencing threat to shut off utility services (“During the last 12 months was there a time when an electric, gas, oil, or water company threatened to shut off services?”), and lack of reliable transportation (“During the past 12 months has a lack of reliable transportation kept you from medical appointments, meetings, work, or from getting things needed for daily living?”).

The HRSN measures were life dissatisfaction (“In general, how satisfied are you with your life?”), lack of social and emotional support (“How often do you get the social and emotional support that you need?”), feeling socially isolated (“How often do you feel socially isolated from others?”), receiving food stamps/Supplemental Nutrition Assistance Program (SNAP) (“During the past 12 months, have you received food stamps, also called SNAP, through the Supplemental Nutrition Assistance Program on an EBT [electronic benefit transfer] card?”), and mental distress (“Stress means a situation in which a person feels tense, restless, nervous or anxious or is unable to sleep at night because their mind is troubled all the time. Within the last 30 days, how often have you felt this kind of stress?”).

An additional measure, cost as a barrier for access to care (“Was there a time in the past 12 months when you needed to see a doctor but could not because of cost?”) was collected as part of the BRFSS core dataset. The prevalence of mammography use among women who reported experiencing these SDOH and HRSNs was analyzed by age group (40–49 years and 50–74 years, respectively) and the variation in having a mammogram with the number of adverse SDOH and HRSNs experienced by state. Because of the low frequency of reporting three or more SDOH and HRSNs, those were grouped together. For each age group, logistic regression models were used to determine which SDOH and HRSNs were significantly related to not having had a mammogram (p<0.05). Each model combined all 39 states and DC and controlled for the other SDOH and HRSNs.

## Results

### Study Population

Among 142,471 female respondents aged 40–74 years, 11,283 (7.9%) declined to answer, had a missing answer, or answered, “don't know/not sure” to at least one of the mammography questions and were excluded from the analysis. An additional 1,737 (1.3%) respondents who reported a personal history of breast cancer, and 11,985 (9.3%) who declined to answer, had a missing answer, or answered “don’t know/not sure” to the question on history of breast cancer were also excluded. The final sample included 117,466 women, representing 82.4% of female respondents aged 40–74 years.

### Mammography Use by Demographic Characteristics

In 2022, the U.S. prevalence of mammography use during the previous 2 years was 59.1% among women aged 40–49 years and 76.5% among those aged 50–74 years (Table 1). Mammography use varied across states, ranging from 44.5% (New Mexico) to 77.8% (South Dakota) among women aged 40–49 years and from 64.0% (Wyoming) to 85.5% (Rhode Island) among those aged 50–74 years. Mammography use among women aged 40–49 years was significantly lower than that among those aged 50–74 years in all states and DC except three (Mississippi, Pennsylvania, and South Dakota). In both age groups, Black women reported the highest prevalence of mammography use within the previous 2 years (65.2% and 82.9% among women aged 40–49 years and 50–74 years, respectively) (Table 2), and mammography use during the previous 2 years increased with increasing income and higher educational attainment. Among women aged 40–49 years and 50–74 years, mammography use was lower among those without health insurance (32.7% and 37.4%, respectively) and those who did not have a personal health care provider (32.7% and 42.2%, respectively) than among women who reported having health insurance (58.7% and 73.9%, respectively) and a personal provider (63.4% and 79.1%, respectively).

**TABLE 1:** Percentage of women aged 40–74 years who reported having had a mammogram within the previous 2 years, by age group and jurisdiction — Behavioral Risk Factor Surveillance System, United States, 2022

Jurisdiction	Age group, yrs % (95% CI)	
	40–49	50–74
**Total**	**59.1 (57.9–60.4)**	**76.5 (75.9–77.1)**
Alabama	62.7 (54.2–70.5)	77.9 (74.4–81.0)
Alaska	53.2 (46.4–59.8)	67.7 (63.5–71.5)
Arizona	54.0 (47.4–60.5)	74.3 (71.3–77.1)
Arkansas	60.0 (53.4–66.3)	74.7 (71.6–77.5)
California	53.4 (47.6–59.2)	76.2 (72.9–79.2)
Colorado	55.2 (50.5–59.8)	70.6 (67.4–73.6)
Connecticut	71.3 (66.0–76.0)	81.9 (79.2–84.2)
Delaware	60.5 (51.4–68.9)	80.2 (76.9–83.1)
District of Columbia	58.5 (50.7–65.9)	77.0 (71.9–81.4)
Florida	60.1 (52.4–67.4)	79.0 (75.7–81.9)
Georgia	62.1 (56.0–67.9)	76.2 (73.2–78.9)
Hawaii	65.3 (59.8–70.3)	78.2 (75.2–81.0)
Idaho	48.7 (43.0–45.4)	67.6 (64.6–70.5)
Illinois	57.3 (50.7–63.7)	72.0 (67.5–76.1)
Indiana	58.0 (53.6–62.2)	77.6 (75.5–79.6)
Iowa	58.0 (52.7–63.1)	79.6 (76.9–82.0)
Kansas	56.3 (51.4–61.2)	73.4 (70.9–75.9)
Kentucky	58.8 (50.5–66.7)	71.9 (67.2–76.1)
Louisiana	68.6 (62.1–74.5)	81.1 (78.3–83.7)
Maine	59.3 (54.0–64.5)	81.5 (79.4–83.4)
Maryland	66.4 (62.0–70.5)	82.9 (81.0–84.6)
Massachusetts	63.5 (58.9–67.8)	84.4 (82.1–86.6)
Michigan	65.9 (60.8–70.6)	77.3 (75.1–79.4)
Minnesota	61.2 (57.5–64.8)	79.5 (77.5–81.4)
Mississippi*	65.8 (58.9–72.2)	72.7 (68.3–76.6)
Missouri	64.3 (58.3–70.0)	74.8 (71.7–77.7)
Montana	53.4 (47.7–59.1)	73.2 (70.1–76.1)
Nebraska	55.5 (49.6–61.2)	77.1 (74.4–79.7)
Nevada	48.7 (38.0–59.6)	69.9 (64.4–74.8)
New Hampshire	65.9 (57.9–73.1)	80.3 (77.7–82.6)
New Jersey	65.3 (59.5–70.8)	75.8 (72.5–78.8)
New Mexico	44.5 (37.4–51.8)	68.1 (64.2–71.8)
New York	66.3 (62.2–70.2)	78.4 (76.0–80.6)
North Carolina	61.7 (54.8–68.2)	80.4 (76.6–83.7)
North Dakota	62.5 (54.5–69.9)	80.8 (77.6–83.7)
Ohio	57.5 (53.2–61.8)	75.5 (73.3–77.6)
Oklahoma	53.1 (47.5–58.7)	68.2 (64.8–71.4)
Oregon	53.8 (48.3–59.1)	77.9 (74.8–80.8)
Pennsylvania*	67.2 (59.1–74.3)	74.5 (69.0–79.4)
Rhode Island	68.5 (61.6–74.7)	85.5 (83.0–87.6)
South Carolina	60.4 (54.9–65.7)	79.3 (77.1–81.3)
South Dakota*	77.8 (67.7–85.4)	71.9 (63.6–78.9)
Tennessee	61.0 (54.0–67.5)	75.5 (71.7–78.9)
Texas	53.6 (47.6–59.4)	73.9 (70.7–76.8)
Utah	53.0 (48.7–57.2)	74.5 (71.7–77.0)
Vermont	48.5 (43.5–53.5)	74.5 (71.8–77.0)
Virginia	63.8 (58.9–68.4)	77.5 (74.9–79.8)
Washington	50.0 (47.1–53.0)	74.2 (72.6–75.8)
West Virginia	60.5 (54.3–66.4)	77.0 (74.1–79.8)
Wisconsin	58.0 (53.2–62.7)	82.4 (80.4–84.2)
Wyoming	48.7 (41.3–56.0)	64.0 (60.3–67.5)

* Overlapping 95% CI between the two age groups.

**TABLE 2:** Percentage of women aged 40–74 years who reported having had a mammogram within the previous 2 years, by age group and sociodemographic characteristics — Behavioral Risk Factor Surveillance System, United States, 2022

Characteristic	Age group, yrs % (95% CI)	
	40–49	50–74
Race and ethnicity*		
American Indian or Alaska Native	54.2 (44.7–63.4)	61.5 (54.8–67.7)
Asian, Native Hawaiian, or other Pacific Islander	54.3 (47.7–60.8)	76.8 (71.8–81.2)
Black or African American	65.2 (62.2–68.2)	82.9 (81.4–84.3)
White	60.4 (59.0–61.7)	76.6 (75.9–77.2)
Hispanic or Latino	54.5 (50.9–58.1)	74.3 (71.6–76.8)
Other race or multiracial	58.5 (51.1–65.4)	66.9 (61.0–72.4)
**Education level**		
Did not complete high school	44.6 (39.5–49.9)	64.9 (61.8–67.9)
Graduated from high school	56.0 (53.0–59.0)	73.5 (72.2–74.8)
Attended college or technical school	56.3 (54.0–58.6)	77.1 (76.1–78.2)
Graduated from college or technical school	67.1 (65.6–68.7)	81.6 (80.8–82.5)
**Annual household income, $**		
<15,000	45.7 (40.6–50.9)	63.1 (59.9–66.2)
15,000 to <35,000	50.7 (47.5–53.9)	69.2 (67.4–71.0)
35,000 to <50,000	55.3 (51.2–59.3)	74.7 (72.8–76.5)
50,000 to <75,000	56.6 (52.9–60.2)	78.0 (76.3–79.5)
≥75,000	65.6 (63.7–67.4)	82.6 (81.6–83.6)
**U.S. Census Bureau region^†^**		
Northeast	65.9 (63.3–68.5)	78.3 (76.7–79.8)
Midwest	59.8 (57.9–61.7)	76.3 (75.3–77.4)
South	59.8 (57.6–61.9)	76.9 (75.9–77.9)
West	52.9 (49.9–55.9)	74.5 (72.9–76.0)
**Metropolitan statistical area**		
Metro	59.6 (58.2–60.9)	77.2 (76.5–77.9)
Nonmetro	56.4 (53.7–59.1)	73.1 (71.8–74.4)
**Has health insurance**		
Yes	58.7 (49.9–66.9)	73.9 (69.8–77.7)
No	32.7 (28.3–37.4)	37.4 (33.6–41.3)
**Has a personal health care provider**		
Yes	63.4 (62.0–64.7)	79.1 (78.5–79.7)
No	32.7 (29.5–36.1)	42.2 (39.0–45.6)

* Persons of Hispanic or Latino (Hispanic) origin might be of any race but are categorized as Hispanic; all racial groups are non-Hispanic.

^†^ https://www2.census.gov/geo/pdfs/maps-data/maps/reference/us_regdiv.pdf

### Association of Mammography Use with Social Determinants of Health and Health-Related Social Needs

Among women aged 50–74 years, median past–2-year mammography use prevalence decreased with increasing number of reported adverse SDOH and HRSNs (Figure) (Supplementary Table, https://stacks.cdc.gov/view/cdc/150461). Among women reporting no adverse SDOH or HRSNs, the median jurisdiction mammography use prevalence was 83.2% (range = 69.6%–91.0%); among those reporting one, two, or 3–11 adverse SDOH or HRSNs, the median state mammography use prevalences were 77.1% (range = 57.9%–89.5%), 73.3% (range = 63.6%–83.5%), and 65.7% (range = 44.8%–83.8%), respectively. The prevalence of reporting more than one adverse SDOH or HRSN among women aged 40–49 years was too low to produce stable estimates.

**FIGURE:**
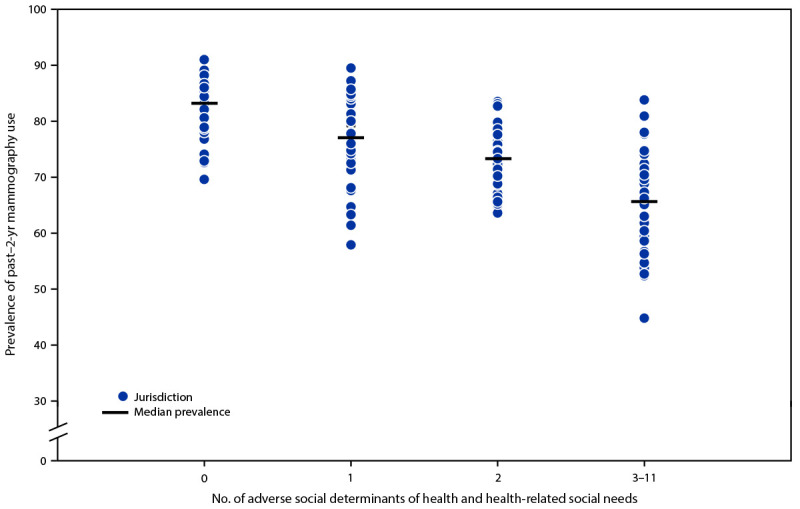
Percentage of women aged 50–74 years who reported having had a mammogram within the previous 2 years, by jurisdiction* and number of reported adverse social determinants of health and health-related social needs — Behavioral Risk Factor Surveillance System, United States, 2022 * Data available for 39 states (Alabama, Alaska, Arizona, California, Connecticut, Delaware, Florida, Georgia, Idaho, Indiana, Iowa, Kansas, Kentucky, Maine, Maryland, Massachusetts, Michigan, Minnesota, Mississippi, Missouri, Montana, Nebraska, Nevada, New Hampshire, New Jersey, New Mexico, North Carolina, Ohio, Oklahoma, Rhode Island, South Carolina, Tennessee, Texas, Utah, Vermont, Washington, West Virginia, Wisconsin, and Wyoming) and the District of Columbia.

In the logistic regression model, among women aged 40–49 years, feeling socially isolated, experiencing lost or reduced employment hours, and reporting cost as a barrier to health care access were significantly associated with not having had a mammogram during the previous 2 years (Table 3). Among women aged 50–74 years, reporting life dissatisfaction, feeling socially isolated, experiencing lost or reduced employment hours, receiving food stamps, lacking reliable transportation, and reporting cost as a barrier to health care access were all associated with not having had a mammogram within the previous 2 years. Among women in both age groups, cost as a barrier to health care access was the measure most strongly associated with not having had a mammogram within the previous 2 years.

**TABLE 3:** Association of adverse social determinants of health and health-related social needs with report of not having had a mammogram* within the previous 2 years among women aged 40–74 years, by age group — Behavioral Risk Factor Surveillance System, United States,^†^ 2022

Adverse SDOH and health-related social needs	Age group, yrs			
	40–49		50–74	
	OR (95% CI)	p-value	OR (95% CI)	p-value
Life dissatisfaction	1.31 (0.94–1.83)	0.11	1.47 (1.20–1.81)	<0.001^§^
Lack of social and emotional support	0.94 (0.77–1.15)	0.52	1.09 (0.96–1.24)	0.18
Feeling socially isolated	1.30 (1.11–1.53)	0.001^§^	1.19 (1.07–1.32)	0.002^§^
Lost or reduced hours for employment	1.35 (1.06–1.71)	0.02^§^	1.20 (1.01–1.43)	0.04^§^
Receiving food stamps (SNAP)	1.17 (0.97–1.42)	0.10	1.29 (1.10–1.52)	0.002^§^
Food insecurity	0.84 (0.67–1.05)	0.12	1.19 (0.99–1.42)	0.06
Housing insecurity	0.99 (0.79–1.25)	0.94	1.02 (0.82–1.26)	0.88
Experiencing threat to shut off utility services	1.23 (0.98–1.56)	0.08	1.24 (0.97–1.58)	0.09
Lack of reliable transportation	1.14 (0.87–1.51)	0.34	1.29 (1.06–1.56)	0.01^§^
Mentally distressed	1.05 (0.88–1.26)	0.59	1.00 (0.86–1.15)	0.96
Cost is barrier to health care access	1.96 (1.55–2.48)	0^§^	2.11 (1.80–2.47)	0^§^

**Abbreviations:** OR = odds ratio; SDOH = social determinants of health; SNAP = Supplemental Nutrition Assistance Program.

* Each logistic regression model controls for the other SDOH and health-related social needs.

^†^ Data available for 39 states (Alabama, Alaska, Arizona, California, Connecticut, Delaware, Florida, Georgia, Idaho, Indiana, Iowa, Kansas, Kentucky, Maine, Maryland, Massachusetts, Michigan, Minnesota, Mississippi, Missouri, Montana, Nebraska, Nevada, New Hampshire, New Jersey, New Mexico, North Carolina, Ohio, Oklahoma, Rhode Island, South Carolina, Tennessee, Texas, Utah, Vermont, Washington, West Virginia, Wisconsin, and Wyoming) and the District of Columbia.

^§^ Significantly associated with not having had a mammogram within the previous 2 years (p<0.05).

## Discussion

In 2022, more than three quarters (76.5%) of women aged 50–74 and more than one half (59.1%) of those aged 40–49 reported having had a mammogram within the previous 2 years. Mammography use varied by state and sociodemographic characteristics. Characteristics related to access to health care (i.e., low income, lack of health insurance, and lack of a personal health care provider) were associated with lower prevalences of mammography use. This finding is consistent with those from previous studies, which have shown associations between lower mammography use and lower educational attainment and income, not having a usual source of health care, and being uninsured (*8*,*9*). Persons who do not have routine health care providers and do not have health insurance might face barriers to receiving health care (*12*).

This analysis incorporated data from a new BRFSS module to explore the relationship between SDOH and HRSNs and mammography use. Individual SDOH and HRSNs, including feeling socially isolated, life dissatisfaction, lost or reduced employment hours, lack of reliable transportation, and cost as a barrier to accessing health care, were associated with not having had a mammogram within the previous 2 years. Cost as a barrier to accessing health care was most strongly associated, which might represent a wide range of factors beyond the cost of health care, including costs for transportation, child care, and taking time off work. Further, mammography use decreased as women experienced an increasing number of adverse SDOH and HRSNs. The impact of these SDOH and HRSNs might have been exacerbated during the COVID-19 pandemic when persons often remained at home, which potentially increased social isolation and job loss (*16*).

Studies have indicated that evidence-based interventions, including programs that provide healthy food options and equitable access to transportation, increase health care adherence and improve health outcomes (*17*). Addressing adverse SDOH and HRSNs might require multicomponent approaches. The White House released an SDOH playbook (*18*) that provides a list of specific measures to improve the social circumstances that adversely affect health. This playbook specifically calls for better understanding and sharing of information on SDOH and HRSNs to guide and improve policy decisions and quality improvement activities, funding of community organizations that focus on specific needs of persons, and coordination of health care with public health and social services. As part of an effort to address some cost concerns, mammograms are available at no cost or low cost through insurance mandates under the Affordable Care Act,^¶^ Medicare,** and CDC’s National Breast and Cervical Cancer Early Detection Program.^††^

In 2024, the Centers for Medicare & Medicaid Services (CMS) implemented a new billing code that allows health care providers to be reimbursed for administering an assessment to identify SDOH and HRSNs.^§§^ CMS also encourages providers to document *International Classification of Diseases, Tenth Revision* codes to capture information on patients’ SDOH and HRSNs in medical records (*19*). Reimbursement, as recognition that these SDOH and HRSNs directly influence health outcomes, could provide incentives to health care providers to perform this assessment during medical visits and link patients to needed social services that address SDOH and HRSNs.

### Limitations

The findings in this report are subject to at least five limitations. First, BRFSS data are based on self-report and are not confirmed by medical record review; this might result in under- or overestimating mammography use. Second, the mammography use question does not distinguish between screening and diagnostic testing, which might lead to overestimating up-to-date mammography use per screening recommendations. Third, this analysis might have included women at high risk for developing breast cancer, for whom USPSTF recommendations do not apply because they require more frequent screening. Fourth, the SDOH and HRSNs assessed in this analysis are not specifically related to mammography use and are not available for all states, which might limit generalizability. Finally, because the BRFSS response rate was 45%, the findings might not be representative of the total adult population.

### Implications for Public Health Practice

In addition to implementing evidence-based interventions to increase mammography use (e.g., client reminders, videos, brochures, flyer, postcards, newsletters, and reducing structural barriers) (*20*), addressing social needs might result in increased mammography use and reduced breast cancer deaths. Health care facilities, providers, and public health programs could consider developing policies and effective practices to conduct risk assessments for adverse SDOH and HRSNs and address SDOH and HRSNs such as cost to access health care, social isolation, lack of reliable transportation, and food insecurity. Addressing SDOH and HRSNs and their drivers might increase the prevalence of receipt of mammography and other preventive health services.

## Footnotes

*
https://uspreventiveservicestaskforce.org/uspstf/index.php/draft-recommendation/breast-cancer-screening-adults

† 45 C.F.R. part 46.102(l)(2), 21 C.F.R. part 56; 42 U.S.C. Sect. 241(d); 5 U.S.C. Sect. 552a; 44 U.S.C. Sect. 3501 et seq.

§ The BRFSS Social Determinants and Health Equity module was administered in Alabama, Alaska, Arizona, California, Connecticut, Delaware, District of Columbia, Florida, Georgia, Idaho, Indiana, Iowa, Kansas, Kentucky, Maine, Maryland, Massachusetts, Michigan, Minnesota, Mississippi, Missouri, Montana, Nebraska, Nevada, New Hampshire, New Jersey, New Mexico, North Carolina, Ohio, Oklahoma, Rhode Island, South Carolina, Tennessee, Texas, Utah, Vermont, Washington, West Virginia, Wisconsin, and Wyoming.

¶
https://www.healthcare.gov/preventive-care-women/

**
https://www.cms.gov/medicare/coverage/preventive-services-coverage

††
https://www.cdc.gov/cancer/nbccedp/

§§
https://www.cms.gov/newsroom/fact-sheets/calendar-year-cy-2024-medicare-physician-fee-schedule-final-rule
